# Supplementary material for: Cerebral hemodynamics in stroke thrombolysis (CHiST) study
Source: PLoS One. 2020 Sep 21;15(9):e0238620. doi: 10.1371/journal.pone.0238620 (PMC7505447; doi:10.1371/journal.pone.0238620)
Supplement: Attachment — Submitted filename: PLOS One ML Rebuttal FINAL 11082020.docx [file pone.0238620.s002.docx]

1^st^ August, 2020

Dear Dr Farag,

Title: Cerebral Hemodynamics in Stroke Thrombolysis (CHiST) Study

(MS. Ref. No.: PONE-D-20-16644)
PLOS ONE

Thank you very much for the opportunity to address the comments of the Reviewers and yourself. We have provided detailed responses below, and additionally tracked associated changes in the manuscript. We trust that these adequately address all the comments, and are grateful for your further consideration of our manuscript.

Journal Requirements:

When submitting your revision, we need you to address these additional requirements.

1. Please ensure that your manuscript meets PLOS ONE's style requirements, including those for file naming. The PLOS ONE style templates can be found at <https://journals.plos.org/plosone/s/file?id=wjVg/PLOSOne_formatting_sample_main_body.pdf> and <https://journals.plos.org/plosone/s/file?id=ba62/PLOSOne_formatting_sample_title_authors_affiliations.pdf>

*Response: Many thanks for these instructions. We confirmed we have read the PLOS ONE’s style requirements carefully and uploaded the revised manuscript, cover letter and figures to the website accordingly.*

2. We note that you have indicated that data from this study are available upon request. PLOS only allows data to be available upon request if there are legal or ethical restrictions on sharing data publicly. For information on unacceptable data access restrictions, please see <http://journals.plos.org/plosone/s/data-availability#loc-unacceptable-data-access-restrictions>.

In your revised cover letter, please address the following prompts:

a) If there are ethical or legal restrictions on sharing a de-identified data set, please explain them in detail (e.g., data contain potentially identifying or sensitive patient information) and who has imposed them (e.g., an ethics committee). Please also provide contact information for a data access committee, ethics committee, or other institutional body to which data requests may be sent.

b) If there are no restrictions, please upload the minimal anonymized data set necessary to replicate your study findings as either Supporting Information files or to a stable, public repository and provide us with the relevant URLs, DOIs, or accession numbers. Please see <http://www.bmj.com/content/340/bmj.c181.long> for guidelines on how to de-identify and prepare clinical data for publication. For a list of acceptable repositories, please see <http://journals.plos.org/plosone/s/data-availability#loc-recommended-repositories>.

We will update your Data Availability statement on your behalf to reflect the information you provide.

*Response: Thank you for the guidance. We confirm there are no restrictions to data access and we have uploaded the minimal anonymized data set as Supporting Information files.*

3.  Thank you for stating the following in the Competing Interests section:

'Professor Thompson G Robinson is the NIHR senior investigator, otherwise, no other
authors have competing interests.'.

Please confirm that this does not alter your adherence to all PLOS ONE policies on sharing data and materials, by including the following statement: "This does not alter our adherence to  PLOS ONE policies on sharing data and materials.” (as detailed online in our guide for authors [http://journals.plos.org/plosone/s/competing-interests](about:blank)).  If there are restrictions on sharing of data and/or materials, please state these. Please note that we cannot proceed with consideration of your article until this information has been declared.

*Response: We have confirmed that ‘Professor Thompson G Robinson is the NIHR senior investigator, otherwise, no other authors have competing interests, this does not alter our adherence to PLOS ONE policies on sharing data and materials’ on revised manuscript (Tracked version: page 1; Clean version: page 1).*

Please include your updated Competing Interests statement in your cover letter; we will change the online submission form on your behalf.

*Response: We confirm we have updated the competing interest statement in our revised cover letter.*

4. Please include a copy of Tables 1 to 6 which you refer to in your text.

*Response: Many thanks for the comment, we have included a copy of Tables 1 to 6 in the revised manuscript.*

5. Please include captions for your Supporting Information files at the end of your manuscript, and update any in-text citations to match accordingly. Please see our Supporting Information guidelines for more information: [http://journals.plos.org/plosone/s/supporting-information](about:blank).

*Response: This can be confirmed as well. We have included our raw data on the Supporting Information Files.*

Additional Editor Comments (if provided):


[Note: HTML markup is below. Please do not edit.]

Reviewers' comments:

Reviewer's Responses to Questions

**Comments to the Author**

1. Is the manuscript technically sound, and do the data support the conclusions?

The manuscript must describe a technically sound piece of scientific research with data that supports the conclusions. Experiments must have been conducted rigorously, with appropriate controls, replication, and sample sizes. The conclusions must be drawn appropriately based on the data presented.

Reviewer #1: Yes

2. Has the statistical analysis been performed appropriately and rigorously?

Reviewer #1: Yes

3. Have the authors made all data underlying the findings in their manuscript fully available?

The [PLOS Data policy](https://eur03.safelinks.protection.outlook.com/?url=http%3A%2F%2Fwww.plosone.org%2Fstatic%2Fpolicies.action%23sharing&data=02%7C01%7Cml376%40leicester.ac.uk%7Cf2f78332b0204cd89bd008d81ddbceb0%7Caebecd6a31d44b0195ce8274afe853d9%7C0%7C1%7C637292177200360518&sdata=U17wprKSqm7aFlXkWnTPmP0Kk3DC2F4vZ4%2BwJylBDXU%3D&reserved=0) requires authors to make all data underlying the findings described in their manuscript fully available without restriction, with rare exception (please refer to the Data Availability Statement in the manuscript PDF file). The data should be provided as part of the manuscript or its supporting information, or deposited to a public repository. For example, in addition to summary statistics, the data points behind means, medians and variance measures should be available. If there are restrictions on publicly sharing data—e.g. participant privacy or use of data from a third party—those must be specified.

Reviewer #1: Yes

4. Is the manuscript presented in an intelligible fashion and written in standard English?

PLOS ONE does not copyedit accepted manuscripts, so the language in submitted articles must be clear, correct, and unambiguous. Any typographical or grammatical errors should be corrected at revision, so please note any specific errors here.

Reviewer #1: Yes

5. Review Comments to the Author

Please use the space provided to explain your answers to the questions above. You may also include additional comments for the author, including concerns about dual publication, research ethics, or publication ethics. (Please upload your review as an attachment if it exceeds 20,000 characters)

Reviewer #1: 1. What are the main claims of the paper and how significant are they for the discipline?
◊ Main purpose of the paper was to conduct a pilot study in intravenous thrombolysis patients to detect changes in cerebral autoregulation during and following successful recanalization at 4 time points in the acute, subacute and chronic stages after an acute ischemic stroke. Transcranial doppler data sets, blood pressure, and endtidal Co2 were monitored.
This pilot study is significant for the discipline given a knowledge gap in understanding the patterns of cerebral autoregulation in various stroke patterns at different time points including the chronic phase and the role the data plays in reperfusion injury after recanalization.
2. Are the claims properly placed in the context of the previous literature? Have the authors treated the literature fairly?
◊ Literature was treated fairly.
3. Do the data and analyses fully support the claims? If not, what other evidence is required?
◊ Data analyses revealed a reduction in BP during intravenous thrombolysis. Reduction in heart rate, reduction in critical closing pressure in the affected and non-affected hemisphere were observed post iv thrombolysis across visits to the 3-month mark. Endtidal CO2 increased during subacute and chronic stages. No changes in cerebral blood velocity, coherence, gain, and Autoregulation Index during the follow-up period.
◊ It would be important to mention whether any or no medication management was applied in the acute phase and in the post-stroke chronic phase while the patients were evaluated. E.g. change in antihypertensive medication, new use of antiarrhythmic drugs (beta blockade for arrhythmia or tachycardia), application or need for supplemental oxygen or anxiolytic, analgesic medication with or without supplemental oxygen, especially when the patient’s airway maintenance was at risk or the patient was agitated. Patients’ altered spontaneous breathing efforts can interfere with systemic blood pressure and cerebral perfusion pressure, as well as fluctuation of etCO2 .

*Response: Many thanks for this important comment. We have confirmed that none of the patients required antihypertensive medication or analgesia during IVT infusion. One patient required supplemental oxygen (1 litre via nasal cannula) during IVT infusion. 4 patients were discharged with additional antihypertensive medication, with two additional patients receiving antihypertensive medication at visit 4 (3 months post stroke symptom onset) (Tracked version: page 10; Clean version: page 9).*

4. PLOS ONE encourages authors to publish detailed protocols and algorithms as supporting information online. Do any particular methods used in the manuscript warrant such treatment? If a protocol is already provided, for example for a randomized controlled trial, are there any important deviations from it? If so, have the authors explained adequately why the deviations occurred?
◊ Authors did follow a detailed protocol, all methods were addressed, no deviations apparent for reviewer. 2 patients were excluded with difficulties to obtain the appropriate TCD windows. This reduced the patient group to 11 study patients and limited the ability to address subgroups.
5. If the paper is considered unsuitable for publication in its present form, does the study itself show sufficient potential that the authors should be encouraged to resubmit a revised version?
◊ I recommend considering this pilot study for publication. The discussion includes reasons why more data is required after this pilot study to better understand the effects of changes in cerebral blood pressure/systemic blood pressure affecting reperfusion injury.
6. Are original data deposited in appropriate repositories and accession/version numbers provided for genes, proteins, mutants, diseases, etc.?
◊ Yes
7. Are details of the methodology sufficient to allow the experiments to be reproduced?
◊ Yes
8. Is the manuscript well organized and written clearly enough to be accessible to non-specialists?
◊ Yes. I do compliment the research team for their candid data review and laying out the limitations of this small pilot study and their interest to address these limitations in a larger cohort to optimize individualized patient care. The manuscript is written clearly, though methods and techniques applied use specific terminology that the non-specialist may not be familiar with, however use of proper terminology cannot be avoided in this context.

6. PLOS authors have the option to publish the peer review history of their article ([what does this mean?](https://eur03.safelinks.protection.outlook.com/?url=https%3A%2F%2Fjournals.plos.org%2Fplosone%2Fs%2Feditorial-and-peer-review-process%23loc-peer-review-history&data=02%7C01%7Cml376%40leicester.ac.uk%7Cf2f78332b0204cd89bd008d81ddbceb0%7Caebecd6a31d44b0195ce8274afe853d9%7C0%7C1%7C637292177200360518&sdata=wMJHOctT0QA%2FjBjzLkZsUMZpdmTii6lZ2Z4o%2FICDQ4E%3D&reserved=0)). If published, this will include your full peer review and any attached files.


If you choose “no”, your identity will remain anonymous but your review may still be made public.


**Do you want your identity to be public for this peer review?** For information about this choice, including consent withdrawal, please see our [Privacy Policy](https://eur03.safelinks.protection.outlook.com/?url=https%3A%2F%2Fwww.plos.org%2Fprivacy-policy&data=02%7C01%7Cml376%40leicester.ac.uk%7Cf2f78332b0204cd89bd008d81ddbceb0%7Caebecd6a31d44b0195ce8274afe853d9%7C0%7C1%7C637292177200370506&sdata=tsfGLy3hgbAdC42LhFHlcSFdPdLZ7fOh0TwTzeQ8ca8%3D&reserved=0).

Reviewer #1: **Yes:** Dorothea Rosenberger MD PhD
